# Acute circulatory deficiency due to endocrinal tumoral manipulation: the pinealoblastoma

**DOI:** 10.11604/pamj.2014.18.168.1983

**Published:** 2014-06-19

**Authors:** Chemchihik Heithem, Ghazi Issaoui, Mejdi Khadraoui, Mohamed Ladib, Walid Naija, Rachid Said

**Affiliations:** 1 AHU anesthésie réanimation CHU sahloul-sousse6tunisie; 2 Neurosurgery service of the UHC Sahloul, Sousse-Tunisia

**Keywords:** Pinealoblastoma, VP shunt, metastasis, circulatory deficiency, serotonin

## Abstract

We rapport the case of a patient presenting intra-abdominal metastasis of a pinealoblastoma, via a ventriculo-peritoneal shunt, confirmed by an anatomo-pathologic exam. The patient presented an acute hydrocephalus secondary to DVP dysfunction. The surgical manipulation of this metastasis had caused an acute circulatory deficiency due to massive serotonin release. In this case we analyze pineal gland physiology and serotonin effect on different systems.

## Introduction

Tumors of the pineal region are rare in adults: 0.4 to 1% of all brain tumors, but more commun in children: 3 to 8% [1, 2, 3]. In humans, the pineal gland is 5 mm long, 1-4 mm thick and weighs about 100 mg in men and women [4]. Pineal parenchymal tumors (PPT) are more rare and represent only 0.3% of all primary tumors of the central nervous system. The size of the tumor is larger in children especially in those younger than 4 years; probably due to greater extensibility and plasticity1. The pinealoblastoma may be associated with the thyroid papillary carcinoma and the colonic polyps [5]. The pinealoblastoma may be also associated with a congenital retinoblastoma. We report the case of a girl presenting a cerebral Pinealoblastoma with abdominal metastasis. The objective of this clinical case is to analyze the physiopathological mechanism of acute insufficiency circulation during surgical manipulation of the abdominal metastasis.

## Patient and observation

Patient Z.B 14-year-old had presented, at the age of 6 years, an intracranial hypertension syndrome. The investigations had concluded for a hydrocephalus secondary to pineal tumor compression. She had a ventriculo-peritoneal derivation (DVP) which was complicated with postoperative meningitis and peritonitis. Bacteriological samples did not isolate germs at LCR and at peritoneal liquid. The evolution was favorable by antibiotics probabilistic treatment based on cefotaxim, Metronidazol and Gentamycin. Then, the patient was lost of view during 6 years.

Six years later, the girl presented a second episode of intracranial hypertension syndrome secondary to the tumor volume increase. She had a tumor incomplete resection. Tumoral anatomopathologic exam objectified a pinealoblastoma, and then the patient had supplementary sessions of radio and chemotherapy.

Two years later, the patient consulted for left iliac fossa (LIF) pain without fever. The abdominal scanography showed mesenteric lymphadenopathy and two intra peritoneal metastasis, the one measuring 78*78*59mm and other one 30*20*20 mm of big axle. 12 hours afterwards, the girl presents a third episode of clinical intracranial hypertension syndrome. The cerebral scanography showed an acute hydrocephalus with a derivation parried in place. The patient was proposed for review of DVP under general anesthesia. Surgical operation consists of two times, a first time for DVP cerebral end review and a second time dedicated for DVP peritoneal end review.

The first surgical time took place without hemodynamic or ventilatory complications and objectifies a permeable valve. The second time showed DVP distal end obstruction by a tumor mass. The patient had a complete resection of this mass. The anatomopathologic analysis objectified a pinealoblastoma and confirmed the hypothesis of intra peritoneal metastasis tumor via DVP.

The manipulation of the tumor mass had caused a circulatory deficiency marqued by an impregnable Sp02, low Pet CO2, a heart rate at 130 flapping / min and an impregnable blood-pressure requiring the administration of Noradrenalin at the dose of 2 mg/h, without important per operative bleeding (300 ml).

The postoperative evolution was marked by an anasarca, a myocardial suffering with troponine at 7 ng/l, hemodynamic unstable state with systolic blood-pressure oscillating between 70 and 210 mm Hg and a sino-atrial tachycardia to 150-170 flapping/min slowed down by amiodarone. 48 h later the patient had become conscious with a correct hemodynamic state authorizing the stopping of catecholamine. Normalization of troponine and extubation at 4 days postoperative. To note that cardiac ultrasound scan was not made. After a week stays in intensive care the girl regained her domicile with a discovery, in post intensive care consultation, of a left lower limb thrombophlebite needing anticoagulation.

## Discussion

The pineal region comprises several anatomical structures that are host to a spectrum of histologically different types of tumors: gliomas, epandymomas, dermoid cysts, meningiomas, cavernomas and metastases. The pineal gland may also present tumors that derive from their own histological components of different behavior. The pineal body parenchyma contains both a large cell, the pineocyte, and a small cell, the pinealoblast: an apparent immature form of the large cell. A pinealocytoma arises from the large cells and a pinealoblastoma from the small cells [6]. Traditionaly, pineal parenchymal tumors (PPT) have been divided, by world health organization (WHO), into 3 types: pinealocytomas, pinealoblastomas and mixed or transitional tumors. This classification is the most commonly accepted. Pinealoblastomas are considered WHO class IV. The electron microscopy plays a useful role in this classification. [7]

The pineal gland parenchyma tumor causes frequently ventricular obstruction. The obstruction may require shunting via a ventriculo-peritoneal shunt for relief of hydrocephalus. The complications of ventriculo-peritoneal shunting for hydrocephalus are numerous and include malfunction, infection, migration of shunt, perforation of viscera, bowel obstruction and metastatic tumor spread [6, 8, 9]. In our case the anatomopathologic exam of the intra-abdominal tumor has confirmed the hypothesis of metastasis tumor by ventriculo-peritoneal shunt.

The pineal bland is a central structure in the cardian system that is innervated by a neural multi-synaptic pathway originating in the suprachiasmatic nucleus (SCN) located in the anterior hypothalamus. The SCN is the major circadian pace maker of the mammalian brain and plays a central role in the generation and regulation of biological rhythms [4].

**Figure 1:**
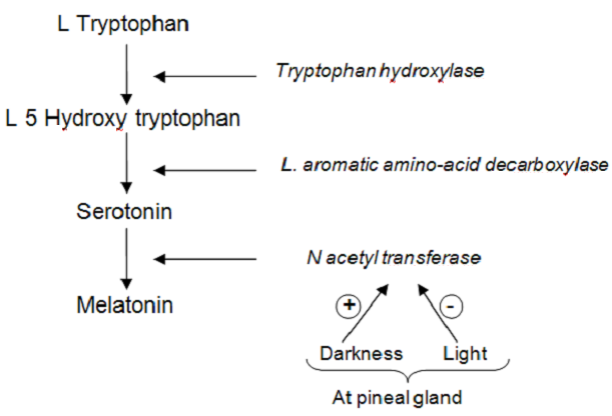
Melatonin synthesis at pineal gland

**Figure 2:**
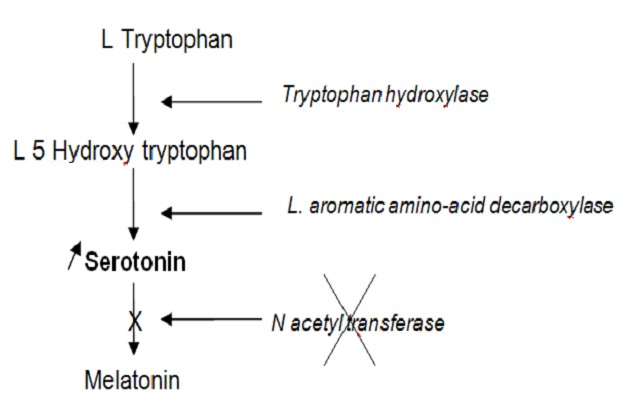
Serotonin accumulation in the pinealoblastoma intra-abdominal metastasis

The biosynthetic pathway of melatonin has been studied thoroughly (Figure 1). L Tryptophan, an essential amine acid, is taken from circulation and hydroxylated then decarboxylated given serotonin (5 HT). The subsequent N acetylation of serotonin by N acetyl transferase is the major regulation step in the biosynthesis: darkness stimulates, light inhibits the synthesis of the N acetyltransferase [10] at pineal gland, leading to a very marked rhythm for melatonin: the maximum being located at night in physiological condition [10]. In our case, the intra-abdominal metastasis tumor produces serotonin but there is no acetylation by N acetyltransferase because this enzyme is synthesized at pineal gland under control of light stimulation. Then there is accumulation of serotonin. Surgical metastasis tumor manipulation can produces very important plasmatic serotonin levels like in carcinoid tumors (Figure 2).

Serotonin receptors are classified into 7 types: 5-HT1, 5-HT2, 5-HT3, 5-HT4, 5-HT5, 5-HT6 and 5-HT7. Serotonin receptors are coupled to G proteins except 5-HT3 receptors which are receptor-channels also called ionotropic receptors. These receptors are localized in brain and in peripheral organs but their distribution are not homogeneous [11]. The signaling pathways to witch these receptors are coupled are known but it is hardly possible to systemize clinical effects corresponding to their stimulation. Serotonin has a positive ionotropic effect and a positive chronotropic action by 5-HT4 receptor stimulation and could take part in the genesis of certain rhythm disorders like in our case. Also, the myocardial ischemia observed in this case can be explicated by coronary vasoconstriction by 5-HT2 effect. Serotonin induces either a vasoconstriction, in particular of renal vessels, a vasodilatation. The response would depend on the preliminary tone of vessels and on their normal or pathological state [11]. Serotonin constricts veins and promotes platelet aggregating then seems to induce venous thromboses [11]. The action on blood pressure is complex, serotonin gives either sever hypotension or hypertension or no modification. The serotonin bronchoconstrictive action was not observed in our case but the patient presented an anasarca due to the important capillary permeability and diarrhea caused by increased intestinal motility by stimulation of 5-HT4 and 5-HT3 receptors [11]. Serotonin is converted into inactive molecules by biotransformation:

1°/ Oxidative deamination of the lateral amino chain by monoamine oxidase (MAO), leading to 5-hydroxy-indol-acetaldehyde which is then oxidized into 5-hydroxy-indol acetic acid (5-HIAA) found in urines in quantities normally lower than 10mg/ 24h. The 5-HIAA was not dosed in our case [11, 12].

2°/ Conjugation by glucuronic acid or sulfate of the hydroxyl group OH in 5 position [11].

## Conclusion

Pinealoblastoma metastatic tumor can be considerated like neuroendocrin tumor which secretes serotonin and caused circulation deficiency, myocardial ischemia, bronchoconstriction, venous thrombosis, anasarca. The anaestisist may be confronted with these problems either in or after surgery.
